# The Impact of Increased Knee Joint Flexion Angle During Loading Response on Mechanical Load of the Knee Joint in Healthy Elderly Individuals: A Cross-Sectional Study

**DOI:** 10.7759/cureus.90117

**Published:** 2025-08-14

**Authors:** Reo Igura, Jun Nakamura, Masaya Anan

**Affiliations:** 1 Clinical Practice and Support, Division of Rehabilitation, Hiroshima University Hospital, Hiroshima, JPN; 2 Physical Therapy, Faculty of Welfare and Health Science, Oita University, Oita, JPN

**Keywords:** gait, knee adduction moment, knee flexion angle, knee flexion moment, motion analysis

## Abstract

Background and Objectives: According to the well-established correlation between knee joint contact force and external knee adduction moment (KAM) during walking, many studies have focused on gait modifications that target changes in the frontal plane, such as foot progression and trunk inclination angles, to decrease KAM. However, recent research has highlighted the relationship between the external knee flexion moment (KFM) and knee joint contact force, highlighting the importance of considering both the KAM and KFM in gait modifications that highlight changes in the sagittal plane. Consequently, the current study aimed to investigate the effects of increasing knee joint flexion angle while walking on knee joint loading.

Methods: This study was a cross-sectional study. 20 healthy older adults each performed two gait conditions - normal walking and walking with a 10-degree increase in the maximum flexion angle, with five trials conducted for each condition.

Results: The results showed that increasing the flexion angle significantly reduced KAM. Specifically, the first peak KAM decreased from 0.59 ± 0.24 to 0.43 ± 0.26 N·m/kg·m (p < 0.001), and the second peak KAM from 0.57 ± 0.20 to 0.45 ± 0.25 N·m/kg·m (p = 0.002). Conversely, the peak KFM increased significantly from 1.5 ± 0.48 to 1.8 ± 0.51 N·m/kg·m (p < 0.001). Furthermore, gait modification involved a straightforward intervention-merely providing verbal instructions to slightly increase knee flexion angle-making it both feasible and practical for older adults at higher risk of developing osteoarthritis of the knee (knee OA).

Conclusions: Ensuring sufficient knee joint flexion angle during the early stance phase may serve as an effective instructional strategy for reducing KAM. Given its simplicity and non-invasive nature, this intervention may have practical potential for use in clinical or community-based gait training programs for older adults at risk of knee OA. However, these findings should be interpreted with caution when generalized to patients with knee OA. Such individuals may have altered muscle activation, increased joint sensitivity, or limited range of motion, which could affect their response to gait modification. Future studies involving longitudinal follow-up or patient-centered outcomes such as pain, function, and adherence will be necessary to evaluate the long-term clinical applicability of this intervention.

## Introduction

Osteoarthritis of the knee (knee OA) is a prevalent degenerative joint disease of the lower limb caused by multiple factors, including aging, obesity, genetic predisposition, and mechanical stress [1]. In Japan, the incidence of knee OA diagnosed using X-ray among individuals aged ≥ 40 years is 25.3 million, of which approximately 8 million suffer from symptomatic knee OA [2]. In rapidly aging nations such as Japan, degenerative diseases are expected to contribute to increasing care demands and social security expenses such as healthcare and nursing care. Therefore, prevention of the onset and progression of knee OA is imperative.

Knee OA causes articular cartilage degeneration and bone alterations around the knee joint, resulting in instability, genu varum, lateral thrust, and gait pain in the lower limbs [3]. Biomechanically, increased contact force in the medial tibiofemoral compartment is a harmful factor responsible for cartilage changes [4]. However, measuring the contact force in the medial tibiofemoral compartment in vivo is often challenging. Therefore, the external knee adduction moment (KAM) is used as a surrogate indicator [5]. It is calculated as the product of the ground reaction force and the perpendicular distance (lever arm) from the knee joint center in the frontal plane. Previous studies have shown that KAM is closely linked to medial knee OA, particularly the first peak KAM, which is associated with cartilage thinning, severity, and pain of medial knee OA [5,6]. Therefore, gait modifications focusing on frontal plane changes are recommended for patients with knee OA with the goal of reducing KAM and slowing disease progression. A previous study reported that increasing the trunk inclination angle, modifying the gait to reduce lateral thrust, and altering the foot progression angle were all effective gait modifications for reducing KAM [7]. While previous studies have primarily investigated gait modification in patients with established knee OA or young adults, limited research has focused on asymptomatic older adults. Given that healthy elderly individuals are at elevated risk for knee OA, investigating biomechanical strategies in this population may offer valuable insights for prevention. The present study is among the first to explore preventive gait modification in healthy older adults to reduce knee joint loading before the onset of symptoms or structural damage.

Although the KAM is considered to be an approximation of the contact force in the medial tibiofemoral compartment, reducing the knee joint load requires consideration of not only the KAM but also the external knee flexion moment (KFM) [6,8]. A previous study reported gait modifications that considered both KAM and KFM in the frontal plane [9]. KFM contributes to articular cartilage loss, and an increase in KFM has been linked to cartilage thinning over five years [6,10]. However, it has been reported that KFM is not a predictive factor for knee OA over a period of two years [11]. This apparent inconsistency may stem from differences in study design, outcome measures (e.g., MRI-based cartilage assessment vs radiographic progression), or follow-up duration. Therefore, comprehensive assessment of both KAM and KFM is essential when evaluating knee joint loading and its implications for disease progression and prevention.

Furthermore, patients with knee OA have been reported to exhibit a decrease in the amount of change in knee joint flexion from the initial contact to peak knee joint angle (knee flexion excursion (KFE)) during the loading response when compared to healthy individuals [12]. KFE plays a critical role in shock absorption and load attenuation during the loading response of gait. Adequate KFE allows the knee joint to dissipate ground reaction forces effectively and facilitates smooth forward progression of the body. In contrast, reduced KFE, as observed in patients with knee OA, may limit shock absorption and lead to increased joint loading, particularly in the medial compartment [13]. This mechanical alteration has been implicated in the progression of knee OA through elevated contact forces and altered neuromuscular control strategies. Promoting an increase in KFE during the loading response may represent a beneficial exercise therapy method for knee OA. In particular, understanding how increased knee flexion affects joint loading in healthy elderly individuals may offer foundational insights for future clinical applications targeting early-stage or at-risk populations. Therefore, this study focuses on healthy older adults to contribute novel knowledge in the context of knee OA prevention and preclinical gait modification.

By confirming these biomechanical relationships in healthy elderly individuals, the current study provides valuable insights into an underrepresented population in previous research. Furthermore, a comprehensive analysis of both the sagittal and frontal plane joint moments provides a more holistic understanding of the effects of gait modification in older adults, which may contribute to the development of preventive strategies for knee OA. While the present study was limited to asymptomatic older adults, the findings may nonetheless offer preliminary insight into the development of preventive strategies for knee OA. Specifically, evaluating biomechanical responses to gait modification in this at-risk but preclinical population allows for the identification of feasible and non-invasive strategies that could potentially be implemented before the onset of joint degeneration or symptoms. This preventive perspective has been underrepresented in prior biomechanical literature, and the present study contributes foundational data to support early-stage intervention approaches.

This study aimed to investigate how increasing KFE during the loading response affects knee joint stress in healthy elderly individuals. We hypothesized that increasing KFE during the loading response would be accompanied by a significant increase in KFM and a decrease in KAM.

## Materials and methods

Participants

The study enrolled 20 healthy elderly individuals aged 60 years or older as participants (Table 1). Participants were included if they met all of the following criteria: (1) age ≥ 60 years; (2) no history of lower-limb surgery; (3) no current or past diagnosis of knee OA; and (4) no current lower-limb pain while walking. Participants were excluded if they had neurological disorders, a history of lower-limb joint replacement or trauma, severe cardiovascular or pulmonary disease, rheumatoid arthritis, or any other conditions that could affect gait performance. This study was conducted in compliance with the regulations of the Ethics Committee of the Faculty of Welfare and Health Science at Oita University (approval no. F200025). 

**Table 1:** Patient characteristics The values (except gender) are represented as mean (SD). BW: Body weight; BH: Body height

Characteristics	
Participants (n)	Female 18, Male 2
Age (years)	69.3 (4.0)
BH (m)	1.52 (0.1)
BW (kg)	50.7 (5.0)
BMI (kg/m^2^)	22.0 (2.2)

Procedure

Kinematic data were collected using a three-dimensional (3D) motion analysis system (VICON, UK) consisting of 10 infrared cameras and reflective markers at a sampling rate of 100 Hz. Kinetic data were collected using eight force plates (AMTI, USA) at a sampling rate of 1,000 Hz. A total of 49 infrared reflective markers were attached to anatomical landmarks throughout the body, including the forehead, occipital bone, anterior superior iliac spine, posterior superior iliac spine, iliac crest, acromion, lateral and medial epicondyles of the humerus, radial and ulnar styloid processes, second metacarpal, greater trochanter, lateral and medial femoral condyles, lateral and medial malleoli, lateral and medial calcaneus, fifth metatarsal, first metatarsal, sternum, xiphoid process, spinous processes of the seventh cervical vertebra, second thoracic vertebra, tenth thoracic vertebra, twelfth thoracic vertebra, and fourth lumbar vertebra on both sides of the body.

**Figure 1:**
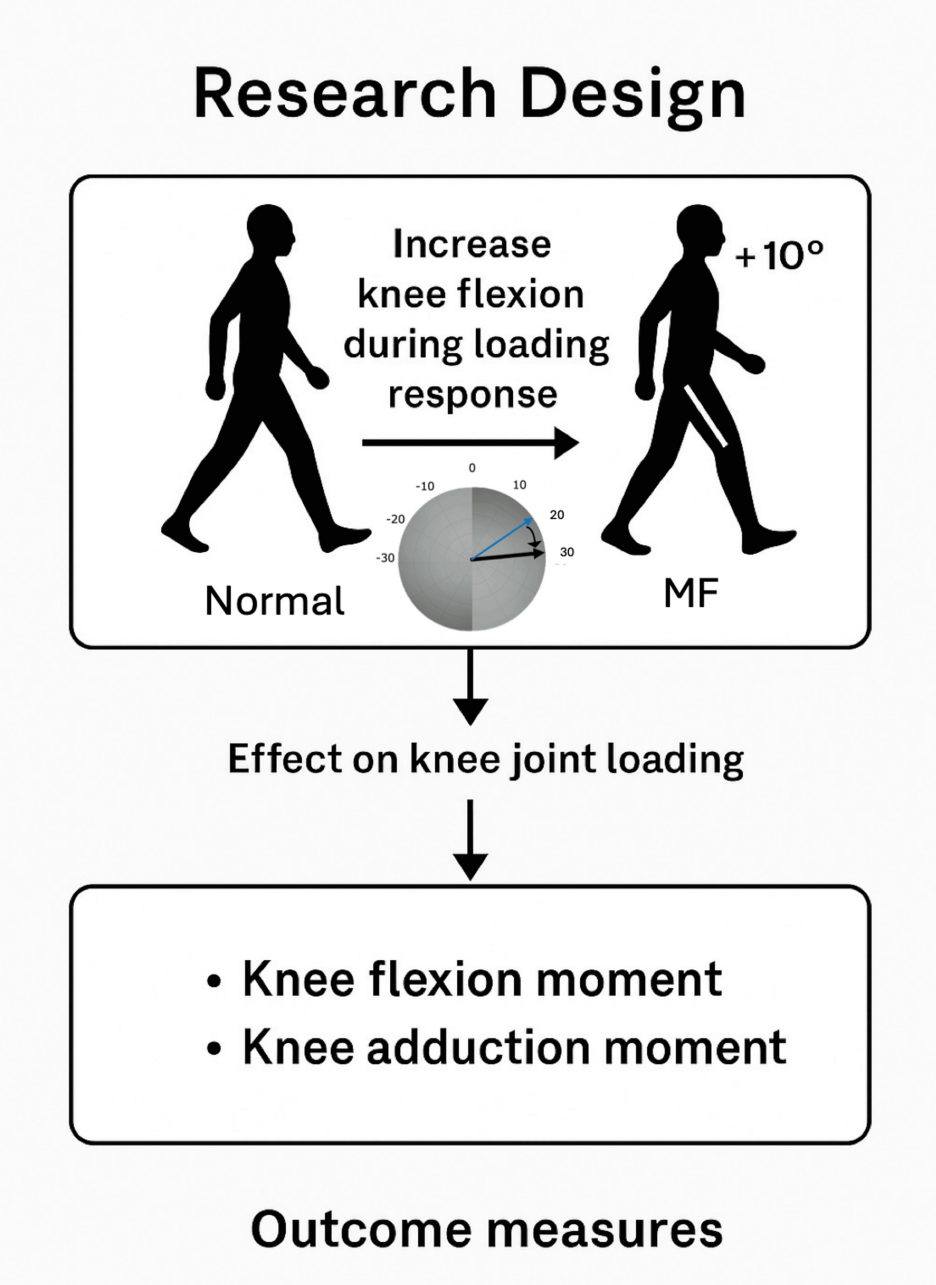
Research design overview

The participants performed five trials of barefoot comfortable gait (N condition) using force plates embedded in the laboratory. The average maximum knee flexion angle during the loading response in the N condition was calculated. The participants then performed a gait task with an increased knee flexion angle of 10° from the average maximum flexion angle of the N condition after practicing for approximately five minutes. In the more flexion (MF) condition, three marker coordinate data points (right greater trochanter, right lateral femoral condyle, and right lateral malleolus) were streamed from Vicon Nexus software into MATLAB R2018a software (MathWorks, USA) in real time. MATLAB was used to calculate and display the knee flexion angle animation based on a previous study (Figure 1) [14]. The examiner continuously monitored real-time visual feedback of the knee flexion angle using a MATLAB-based animation, which was generated from marker data streamed from the Vicon system. This allowed the examiner to confirm whether the participant achieved the target increase in knee flexion. If the target angle was not sufficiently achieved, the trial was judged as unsuccessful and repeated until five successful trials were obtained. Participants did not receive any visual feedback during data collection, and all guidance was provided verbally to avoid influencing their natural gait patterns.

Data analysis

For data processing, the Vicon Nexus software was used to calculate the data from the collected marker coordinates and floor reaction forces. The analysis was limited to the right entire stance phase. This was defined as the time point at which the vertical component of the ground reaction force exceeded 10 N at the initial contact and dropped below 10 N at toe-off. Only the right stance phase was analyzed to ensure consistency across participants and to avoid data redundancy. As the participants were healthy older adults with no known musculoskeletal or neurological impairments, no substantial functional asymmetry was expected. Data analysis was conducted for each condition.

The collected data were filtered using a Butterworth filter with a cutoff frequency of 6 Hz for marker coordinates and 20 Hz for ground reaction forces. The spatial coordinate system was established with the X-axis representing the lateral direction (right: +), the Y-axis representing the anteroposterior direction (anterior: +), and the Z-axis representing the vertical direction (upward: +). The Vicon Nexus software was used to calculate various parameters: KFE, first peak KAM (*fp*KAM), second peak KAM (*sp*KAM), peak KFM (*p*KFM) at the stance phase, KAM and KFM angular impulses during the stance phase, peak lever arm values in the sagittal and frontal planes, first peak vertical ground reaction force (*fp*VGRF), second peak VGRF (*sp*VGRF), foot progression angle from the foot segment, trunk inclination angle from the thoracic segment, and gait parameters (gait speed, step length, cadence, and step width). The stance phase, which served as the analysis interval, was normalized to 100%, and the mean value obtained from five trials for each condition was used as the representative value. The joint moments were normalized using body weight (BW) and body height (BH), while the lever arms were normalized using BH. The VGRF was normalized to BW.

Statistical analysis

Statistical analyses were conducted using IBM SPSS Statistics 23 software (IBM Japan, Japan). The normality of the data was assessed using the Shapiro-Wilk test. If the data were normally distributed, paired t-tests were used; otherwise, the Wilcoxon signed-rank test was applied. Subsequently, an analysis of covariance (ANCOVA) was conducted using KFE, *fp*KAM, *sp*KAM, *p*KFM, KAM angular impulse, KFM angular impulse, *fp*VGRF, and *sp*VGRF as dependent variables, gait condition as a fixed factor, and gait speed as a covariate to adjust for the effect of gait speed. The level of significance was set at p = 0.05.

A post-hoc power analysis was conducted using G*Power to assess the adequacy of the sample size. With a sample size of 20, *α* = 0.05, and an assumed effect size of Cohen’s *d* = 0.65, the statistical power (1 − *β*) exceeded 0.80 for the primary outcomes (e.g., KAM and KFM). Although some secondary outcomes showed slightly lower power, the sample size was considered sufficient for evaluating the study’s main hypotheses.

## Results

Participants had a significantly lower gait speed (p < 0.001), lower cadence (p < 0.001), and higher step width (p = 0.001) in the MF condition than in the N condition (Table 2). However, no significant differences were observed between the two conditions in the foot progression angle (p = 0.246), trunk inclination angle (p = 0.165), or step length (p = 0.105).

**Table 2:** Comparison of knee flexion parameters between conditions The values are represented as mean (SD). Test statistic values are reported as t from paired t-tests. MF: More flexion; KFE: Knee flexion excursion

	Normal	MF	Effect size	Power	Test statistic	p
Peak knee flexion angle (deg)	23.6 (7.7)	37.8 (7.0)	2.31	1.00	t = -10.3	< 0.001
KFE (deg)	10.1 (3.7)	18.0 (4.7)	1.55	1.00	t = -6.95	< 0.001

**Figure 2:**
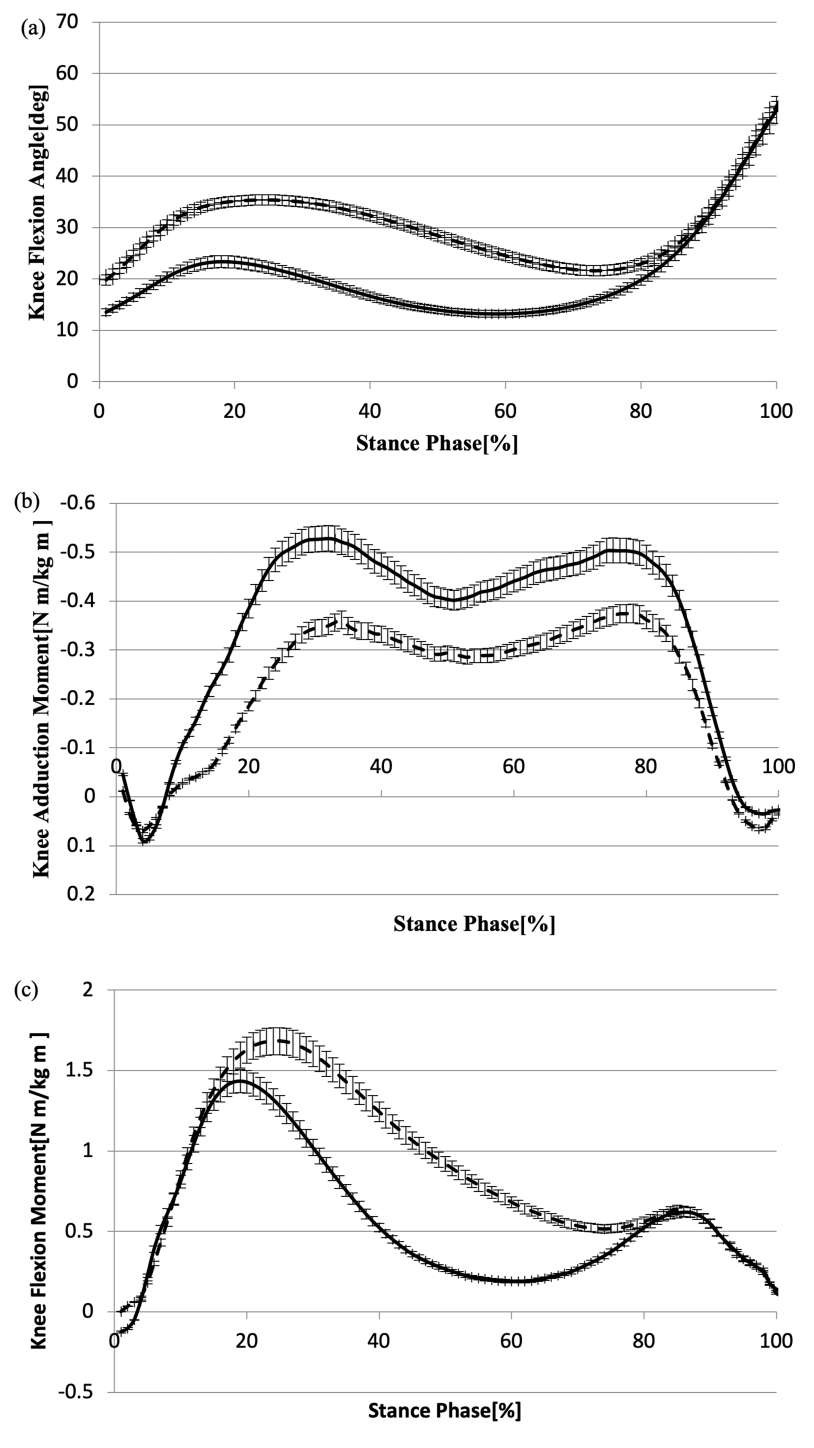
Change in (a) knee joint angle, (b) KAM, and (c) KFM during stance phase The dashed line represents the MF condition, and the solid line represents the N condition. KAM: Knee adduction moment; KFM: Knee flexion moment; MF: More flexion

In the MF condition, there was a significant increase in both the peak knee flexion angle during the loading response (p < 0.001) and KFE (p < 0.001) when compared to the N condition (Figure 2, Table 3). However, the ANCOVA results indicated that the effect of gait speed on KFE was not significant (standardized coefficient (*β*): 6.578, 95% confidence interval (CI): -4.334 to 17.489, p = 0.230).

**Table 3:** Comparison of joint kinetics and related parameters between conditions The values are represented as mean (SD). Test statistic values are reported as t for paired t-tests and Z for Wilcoxon signed-rank tests. MF: More flexion; *fp*KAM: First peak knee adduction moment; *sp*KAM: Second peak knee adduction moment; *p*KFM: Peak knee flexion moment; *fp*VGRF: First peak vertical ground reaction force; *sp*VGRF: Second peak vertical ground reaction Force; BW: Body weight; BH: Body height

	Normal	MF	Effect size	Power	Test statistic	p
*fp*KAM (N·m/kg·m)	0.59 (0.24)	0.43 (0.26)	1.24	1.00	Z = -3.62	< 0.001
*sp*KAM (N·m/kg·m)	0.57 (0.20)	0.45 (0.25)	0.80	0.92	t = -3.58	0.002
*p*KFM (N·m/kg·m)	1.5 (0.48)	1.8 (0.51)	0.96	0.98	t = -4.30	< 0.001
KAM angular impulse (N·m·s/kg·m)	0.36 (0.14)	0.27 (0.15)	1.33	1.00	Z = -3.58	< 0.001
KFM angular impulse (N·m·s/kg·m)	0.58 (0.21)	0.87 (0.29)	1.64	1.00	Z = -3.88	< 0.001
Lever arm in the frontal plane (% BH)	36.0 (9.1)	32.2 (8.4)	0.67	0.79	Z = -2.54	0.011
Lever arm in the sagittal plane (%BH)	59.6 (11.0)	77.9 (13.5)	1.74	1.00	t = -7.77	< 0.001
*fp*VGRF (% BW)	16.5 (1.6)	16.5 (1.5)	0.01	0.05	t = 0.06	0.954
*sp*VGRF (% BW)	16.4 (1.4)	15.8 (1.3)	0.45	0.46	Z = -1.87	0.062

The participants had significantly higher *p*KFM (p < 0.001), higher KFM angular impulse (p < 0.001), and higher lever arm in the sagittal plane (p < 0.001). They had lower *fp*KAM (p < 0.001), *sp*KAM (p = 0.002), KAM angular impulse (p < 0.001), and lever arm in the frontal plane (p = 0.011) in the MF condition than in the N condition (Table 4). However, no significant differences were observed between the two conditions for *fp*VGRF (p = 0.954) and *sp*VGRF (p = 0.062). Additionally, ANCOVA results indicated that gait speed only affected *p*KFM (*β*: 1.872, 95% CI: 0.734 to 3.011, p = 0.002) and *fp*VGRF (*β*: 6.531, 95% CI: 3.127 to 9.936, p < 0.001). On the other hand, *fp*KAM (*β*: 0.611, 95% CI: -0.009 to 1.231, p = 0.053), *sp*KAM (*β*: 0.389, 95% CI: -0.192 to 0.971, p = 0.183), KAM angular impulse (*β*: -0.032, 95% CI: -0.698 to 0.034, p = 0.074), KFM angular impulse (*β*: 0.406, 95% CI: 0.236 to 1.048, p = 0.208), lever arm in the frontal plane (*β*: -9.693, 95% CI: -32.485 to 13.099, p = 0.394), lever arm in the sagittal plane (*β*: 28.520, 95% CI: -2.415 to 59.455, p = 0.070), and *sp*VGRF (*β*: 1.695, 95% CI: -1.760 to 5.149, p = 0.327) were not affected by gait speed.

**Table 4:** Gait parameter The values are represented as mean (SD). Test statistic values are reported as t for paired t-tests and Z for Wilcoxon signed-rank tests. MF: More flexion

	Normal	MF	Effect size	Power	Test statistic	p
Gait speed (m/s)	1.2 (0.1)	1.0 (0.1)	1.42	1.00	t = 6.37	< 0.001
Cadence (steps/min)	124.6 (10.7)	111.7 (10.0)	1.46	1.00	Z = -3.77	< 0.001
Step length (m)	0.56 (0.07)	0.61 (0.13)	0.38	0.36	t = -1.70	0.105
Step width (m)	0.12 (0.06)	0.17 (0.08)	0.73	0.85	Z = -3.32	0.001
Foot progression angle (deg)	11.2 (3.1)	11.9 (3.6)	0.21	0.14	t = 1.20	0.246
Trunk inclination angle (deg)	1.2 (1.9)	0.41 (2.8)	0.32	0.28	t = -1.44	0.165

## Discussion

In this study, we examined the alterations in mechanical stress on the knee joint by augmenting the knee flexion angle during the loading response of gait in healthy elderly individuals. Our findings revealed a significant increase in knee joint load in the sagittal plane, while a significant decrease in knee joint load was observed in the frontal plane as the knee flexion angle increased, supporting our hypothesis.

Initially, the maximum knee flexion angle and KFE during the loading response were significantly higher in the MF condition than in the N condition. This finding indicates that gait modification under MF conditions is possible in healthy elderly individuals. Furthermore, *p*KFM, KFM angular impulse, and sagittal lever arm were significantly higher in the MF condition compared to the N condition, but *fp*VGRF did not differ significantly between the two conditions. In other words, the KFM increased as the knee flexion angle during the loading response increased and the lever arm length in the sagittal plane increased. A previous study reported a positive correlation between peak knee flexion angle and peak KFM in the loading response, and the results of this study were similar to previous studies [15]. 

In the MF condition, *fp*KAM, *sp*KAM, and KAM angular impulses experienced a significant decrease compared to the N condition, whereas step width increased significantly. This suggests that increasing the step width reduced the lever arm length in the frontal plane by shifting the center of gravity to the stance side and bringing the ground reaction force vector closer to the center of the knee joint. Previous studies reported a similar reduction in KAM owing to an increase in step width, although they targeted healthy young individuals, and another study indicated a correlation between gait speed and KAM in knee OA patients and healthy elderly individuals [16-18]. However, the results showed no effect on walking speed or KAM in healthy elderly subjects. According to a study by Akimoto et al., patients with knee OA exhibit significant variability in the gait cycle compared to healthy individuals, and gait cycle variability was reported to have a positive correlation with gait speed in the 5-meter gait test [19]. However, because the participants in this study were healthy elderly individuals, we believe that there was no correlation between gait speed and KAM. But given that the study participants were healthy elderly individuals, it is unlikely that gait speed significantly influenced the KAM in this cohort. Despite this, gait speed was used as a covariate in the statistical analysis because it is a widely recognized confounding variable with a significant impact on joint kinetics. Although other parameters, such as step width, were recorded and showed significant differences between conditions, they were not included as covariates because of the limited sample size (n = 20) to minimize the risk of model overfitting. Because there were no significant differences in the foot progression angle or trunk inclination angle, they had no effect on KAM reduction in the MF condition. 

Gait modifications intended to decrease KAM during gait tend to increase KFM owing to an increase in KFE [20]. Similar results were obtained in our study, which was the first to investigate the effect of sagittal plane gait modifications on contact force in the medial tibiofemoral compartment associated with both KAM and KFM [7,21]. Previous studies that evaluated KAM and KFM as risk factors for structural OA progression over time discovered that peak KFM was not a predictor of disease progression [11]. However, other studies have highlighted potential adverse effects associated with elevated KFM. Zeighami et al. demonstrated that KFM is a strong predictor of medial contact force during gait, suggesting that increased KFM may elevate mechanical stress on the medial compartment [22]. Furthermore, Chehab et al. reported that higher KFM was associated with greater cartilage thinning over time in individuals with knee OA [6].

These findings underscore the importance of interpreting KFM-related changes with caution. Although increased knee flexion elevated KFM, its clinical relevance remains uncertain in populations with joint impairment. Future studies should, therefore, carefully examine both the potential advantages and unintended consequences of sagittal plane loading in gait interventions. Furthermore, KFM decreases as knee OA progresses, and reduced knee flexion during the loading response may increase the co-contraction of the periprosthetic knee muscles, resulting in increased contact force in the medial tibiofemoral compartment. Furthermore, previous studies have investigated KFM in frontal plane gait retraining for pathological populations with knee OA and reported that increased knee flexion can improve medial knee joint load distribution and gait efficiency [23]. These findings suggest that the results observed in healthy elderly individuals could contribute to future clinical applications in OA management. Therefore, gait retraining aimed at increasing the knee flexion angle during the loading response in patients with knee OA may help reduce mechanical stress on the knee joint in the frontal plane.

Gender differences may also affect knee joint loading. The predominance of female participants in this study may have affected our results. Although the joint moments were normalized for body mass and height, previous studies have suggested that gender differences in knee joint biomechanics may persist even after normalization. Anatomical factors such as greater pelvic width and increased Q-angle in female participants may result in different lower extremity alignments, potentially affecting joint loading patterns [24]. Although KAM has not consistently shown gender-related differences, several studies have reported that women tend to exhibit greater KFM during the loading response phase of gait [25,26]. This could be attributable to differences in quadriceps strength, neuromuscular control, and kinematic strategies between genders [26,27]. Therefore, the potential influence of gender on knee-joint loading should be carefully considered when interpreting the results of the present study. In this study, the participants were instructed to increase their knee flexion angle during the loading response without any restrictions on their gait parameters, such as gait speed. Therefore, highlighting the importance of maintaining a sufficient knee flexion angle during the loading response in healthy elderly individuals could be an effective method for decreasing KAM. However, it must be considered that mechanical stimulation is necessary for articular cartilage and that habitual underloading is also detrimental to cartilage health [24].

In addition, gait modification in a population that has not yet presented with a pathological condition may affect joint health. Furthermore, according to Besier et al., deep knee flexion activities such as squatting substantially increase patellofemoral joint reaction forces, which may contribute to pain in individuals with knee OA [28]. This indicates that gait modifications might not be suitable for all patients, particularly those with advanced disease or severe pain. Therefore, the applicability and safety of increasing knee flexion in symptomatic individuals should be carefully considered when extending these interventions to the clinical population. While the present study was limited to asymptomatic older adults, the findings may nonetheless offer preliminary insight into the development of preventive strategies for knee OA. The observed reduction in KAM through a simple verbal instruction to increase knee flexion during the loading response may inform future interventions aimed at modulating medial knee joint loading before structural damage or pain occurs. Such approaches could potentially be incorporated into community-based exercise programs or clinical gait training, pending further validation in longitudinal and symptomatic populations.

This study has several limitations. First, while verbal instruction successfully increased the knee flexion angle in healthy elderly individuals, it remains unclear to what extent this modification effectively reduces KAM or whether further increases in knee flexion would result in additional load reductions. Moreover, given the observed individual variability in knee flexion angle under normal gait conditions, future research should determine which individuals will benefit the most from this intervention.

Second, the participants were healthy older individuals. Therefore, the biomechanical responses observed in this study may differ from those of individuals with knee OA, who frequently experience pain, reduced mobility, or joint deformities. Increased knee flexion in these patients can cause discomfort or instability. Although the verbal instruction used here is a practical and easily implemented strategy, its clinical applicability, particularly for individuals with symptomatic knee OA, requires careful consideration. Therefore, additional training and supervision are necessary to ensure safety. Future studies should evaluate the feasibility, safety, and training requirements of this intervention in clinical populations.

Third, this study used a within-subjects design without randomization or a control group. Although this design reduces inter-individual variability, it limits the findings’ generalizability. Furthermore, because the gait conditions were applied in a fixed order - normal walking followed by the increased knee flexion condition - the potential for order effects, such as learning or fatigue, cannot be excluded. This may have influenced gait performance or joint kinetics in the second condition. In future studies, counterbalancing or randomizing the trial order is recommended to minimize such bias. In addition, the intervention was applied acutely, and long-term feasibility, such as habituation, adherence, and real-world implementation, was not assessed.

Fourth, the study focused exclusively on surrogate biomechanical measures (KAM and KFM) without evaluating clinical outcomes such as pain, cartilage integrity, or long-term functional capacity. Although these kinetic variables are informative, future studies should include clinical assessments to evaluate their therapeutic relevance more comprehensively. Furthermore, gait quality, comfort, perceived effort, and balance were not assessed. Such factors may influence the safety and acceptability of gait modification, particularly in older individuals, and should be incorporated into future investigations.

Fifth, although the gait speed was statistically adjusted using ANCOVA, significant differences in walking speed remained between the conditions. This raises concern about the potential confounding influence of gait speed on the observed kinetic variables, especially *p*KFM and *fp*VGRF, which were shown to be significantly affected by speed in the ANCOVA results. While ANCOVA is a commonly used approach to statistically adjust for such differences, it may not fully eliminate speed-related effects, particularly in studies with small sample sizes or multiple biomechanical outcomes. Therefore, the interpretation of differences in joint kinetics should be made with caution. In future studies, implementing protocols that control or standardize walking speed-such as using treadmill-based trials or real-time feedback-may enhance internal validity and reduce speed-related confounding.

Sixth, the participant cohort consisted predominantly of women (90%). Although gender-related differences in joint biomechanics were discussed in the main text, this imbalance may limit the generalizability of the findings, particularly regarding outcomes such as KFM. Prior research has reported that women often exhibit higher KFM during gait compared to men, which may have influenced the results. Future studies with a more gender-balanced sample are needed to validate these findings across sexes.

## Conclusions

Ensuring sufficient knee joint flexion angle during the early stance phase may serve as an effective instructional strategy for reducing KAM. Given its simplicity and non-invasive nature, this intervention may have practical potential for use in clinical or community-based gait training programs for older adults at risk of knee OA. However, these findings should be interpreted with caution when generalized to patients with knee OA. Such individuals may have altered muscle activation, increased joint sensitivity, or limited range of motion, which could affect their response to gait modification. Moreover, as this study focused on the immediate biomechanical effects in asymptomatic older adults, further research is required to evaluate long-term outcomes, such as symptom improvement or structural progression in patients with knee OA. Future studies involving longitudinal follow-up or patient-centered outcomes (e.g., pain, function, and adherence) will be necessary to assess the clinical applicability and sustainability of this intervention in real-world settings.
